# Smoking prevalence in Medicaid has been declining at a negligible rate

**DOI:** 10.1371/journal.pone.0178279

**Published:** 2017-05-25

**Authors:** Shu-Hong Zhu, Christopher M. Anderson, Yue-Lin Zhuang, Anthony C. Gamst, Neal D. Kohatsu

**Affiliations:** 1 Department of Family Medicine and Public Health, University of California, San Diego, La Jolla, California, United States of America; 2 Moores Cancer Center, University of California, San Diego, La Jolla, California, United States of America; 3 Department of Mathematics, University of California, San Diego, La Jolla, California, United States of America; 4 Department of Health Care Services, Sacramento, California, United States of America; University College London, UNITED KINGDOM

## Abstract

**Background:**

In recent decades the overall smoking prevalence in the US has fallen steadily. This study examines whether the same trend is seen in the Medicaid population.

**Methods and findings:**

National Health Interview Survey (NHIS) data from 17 consecutive annual surveys from 1997 to 2013 (combined *N* = 514,043) were used to compare smoking trends for 4 insurance groups: Medicaid, the Uninsured, Private Insurance, and Other Coverage. Rates of chronic disease and psychological distress were also compared.

**Results:**

Adjusted smoking prevalence showed no detectable decline in the Medicaid population (from 33.8% in 1997 to 31.8% in 2013, trend test *P* = 0.13), while prevalence in the other insurance groups showed significant declines (38.6%-34.7% for the Uninsured, 21.3%-15.8% for Private Insurance, and 22.6%-16.8% for Other Coverage; all *P*’s<0.005). Among individuals who have ever smoked, Medicaid recipients were less likely to have quit (38.8%) than those in Private Insurance (62.3%) or Other Coverage (69.8%; both *P*’s<0.001). Smokers in Medicaid were more likely than those in Private Insurance and the Uninsured to have chronic disease (55.0% vs 37.3% and 32.4%, respectively; both *P*’s<0.01). Smokers in Medicaid were also more likely to experience severe psychological distress (16.2% for Medicaid vs 3.2% for Private Insurance and 7.6% for the Uninsured; both *P*’s<0.001).

**Conclusions:**

The high and relatively unchanging smoking prevalence in the Medicaid population, low quit ratio, and high rates of chronic disease and severe psychological distress highlight the need to focus on this population. A targeted and sustained campaign to help Medicaid recipients quit smoking is urgently needed.

## Introduction

In recent decades, smoking prevalence in the US has steadily decreased for a number of reasons: increased public awareness of the health consequences of smoking and secondhand smoke exposure, widespread adoption of higher tobacco taxes, policies limiting where smoking is allowed, and a wide range of effective smoking cessation treatments.[1,2] The decline in smoking prevalence has improved population health and reduced health care costs nationwide.[1]

Despite the encouraging overall progress, large demographic disparities in tobacco use remain and may even be growing larger over time, suggesting that certain subpopulations are not benefitting fully from the general trend toward tobacco-free living. For example, smoking prevalence is consistently higher in the low-income population, and decreasing more slowly, than in the general population.[1,3,4]

The present study focuses on smoking behavior in a subset of the low-income population: those insured by Medicaid. Medicaid is the primary means-tested form of health insurance in the US, and is funded jointly by the federal and state governments. It has been observed that smoking prevalence for Medicaid recipients is nearly double that of the general population.[5] Smoking has an enormous impact on both the health of the insured and on the cost of operating the program. The 2014 Surgeon General’s Report estimates that 15.2% of Medicaid costs are attributable to smoking, or about $40.1 billion in 2010.[1,6] With the expansion of Medicaid under the Affordable Care Act (ACA), it has been estimated that this figure could rise to more than $75 billion by 2016.[5] Clearly, governments have strong interests––both humanitarian and financial––in reducing the rate of smoking among Medicaid beneficiaries. These interests are certainly reflected in the ACA goals of improving health care quality and improving health outcomes while reducing the cost of care.[7]

Recognizing the important role of state Medicaid programs in reducing the national toll of tobacco-related death and disease, various federal agencies have advocated for improvements in how programs deal with tobacco use by their members. The US Public Health Service recommends that all insurers, including Medicaid, provide comprehensive coverage for effective tobacco dependence treatments, including both medication and counseling.[8] Healthy People 2020 sets a goal of increasing comprehensive Medicaid cessation coverage throughout the US.[9] The US Department of Health and Human Services, in a first-of-its-kind strategic action plan for tobacco control, also advises provision of a comprehensive benefit and for the elimination of treatment barriers such as copayments and limited treatment courses.[10] The Centers for Disease Control and Prevention (CDC) 6|18 Initiative recommends smoking cessation to health care purchasers and providers as one of six interventions that can improve health and reduce health care costs.[11,12] The Affordable Care Act itself forbids states from excluding Food and Drug Administration (FDA)-approved cessation medications in their traditional Medicaid coverage, and requires Medicaid expansion states to cover cessation services with no cost-sharing for their newly eligible beneficiaries.[7]

The present study aims to assess progress made toward reducing the rate of smoking by Medicaid recipients prior to expansion. In order to accomplish this, the study examines National Health Interview Survey (NHIS) data collected from 1997, when the survey was redesigned, to 2013, just before the expansion. The NHIS is the most comprehensive source of information about the health of the US population, and because it assesses health insurance coverage as well as smoking behavior it is ideal for the purpose of analyzing patterns of tobacco use by insurance status.[13] This study compares the smoking behavior of those with and without Medicaid coverage. It also compares them with respect to the prevalence of common chronic diseases that may be caused or aggravated by smoking. To explore further the factors that may contribute to increased difficulty with quitting, the study also examines differences in rates of psychological distress. By understanding the patterns of tobacco use and cessation in the Medicaid population, stakeholders will be better equipped to help Medicaid members quit.

## Methods

### Participants

NHIS is a continuously administered in-person household interview survey conducted by the CDC’s National Center for Health Statistics.[13] NHIS data were selected starting in 1997, when the survey underwent a major redesign. 2013 was chosen as the final study year because in 2014 the Affordable Care Act (ACA) began sharply expanding Medicaid. The combined sample size of the 17 annual surveys used in the study is 514,043. Survey methods can be found at http://www.cdc.gov/nchs/nhis/methods.htm.

Informed consent was not required because the data was analyzed anonymously. All research activity for this study was approved by the University of California, San Diego Human Research Protections Program (#140821).

### Measures

#### Measures of smoking and quitting

Ever smokers are those who have smoked 100 or more cigarettes in their lifetimes. Current smokers are those ever smokers who smoked cigarettes every day or some days at the time of survey. Smoking prevalence is defined as the percentage of adults who are current smokers at the time of the interview. The quit ratio is the percentage of ever smokers who report at the time of the survey that they no longer smoke.

The quit attempt rate is the percentage of smokers who made a quit attempt in the previous 12 months. A quit attempt is an intentional cessation of tobacco use for at least 24 hours. The 3-month quit rate is the percentage of smokers who tried to quit smoking in the last 12 months and succeeded in quitting for at least 3 months at the time of survey.

#### Measure of insurance status

Health insurance status in the NHIS data is coded in four categories: Medicaid, Uninsured, Private Insurance, and Other Coverage. Medicaid includes those who do not have private insurance but who do have Medicaid or another state-sponsored health plan. Uninsured includes those who have no health insurance, who only have the Indian Health Service, or who only have a plan that pays for a single type of service such as dental care. Private Insurance includes those with a comprehensive insurance plan provided by an employer, purchased directly, or obtained through local or community programs. Other Coverage includes those with Medicare, a military health plan such as TRICARE, VA, or CHAMPVA, or government-provided coverage other than Medicaid.[14]

#### Measures of physical and psychological health

Chronic diseases studied include hypertension, heart disease, stroke, emphysema, asthma, cancer and diabetes. Subjects are considered to have chronic disease if they report that a health professional ever told them they have one of the above conditions. Heart disease is a composite of four conditions assessed individually in the survey: coronary heart disease, angina pectoris, heart attack, and other heart condition or disease.

Subjects are considered to be experiencing severe psychological distress if they score 13 or higher on an NHIS question that is based on the Kessler Psychological Distress Scale or K6.[15] Subjects are asked, “During the past 30 days, about how often did you feel (1) nervous, (2) hopeless, (3) restless or fidgety, (4) so sad or depressed that nothing could cheer you up, (5) that everything was an effort, [or] (6) worthless?” Responses are coded as 4 = all of the time, 3 = most of the time, 2 = some of the time, 1 = a little of the time, or 0 = none of the time.

### Statistical analysis

For each individual survey year, all analyses were weighted to adjust for the unequal probability of selection in sampling. For this purpose, the weights provided by NHIS data set for each survey year were used.[16] When comparing the prevalence rate over time, we standardized the data from the 1998 and later surveys to the demographic composition of the 1997 survey. This was to ensure that apparent changes in population smoking behavior were not due simply to demographic changes over time. When the data from 1997 to 2013 were combined in the same analysis (e.g., annual quit attempt rate by insurance coverage), the weights were adjusted to the sum of the observed sample size of each survey.

When testing for a trend of changing smoking prevalence over time, we used two methods: a liberal test in which a simple linear trend test was performed without considering increased type I error due to comparisons over multiple years, and a conservative test in which 99.7% confidence intervals, instead of 95% confidence intervals, were computed for each survey. The stricter confidence intervals were used to adjust for multiple comparisons, so as to retain an overall 95% family-wise error rate for the 17 surveys.

The quit ratio was analyzed for the last year of the survey examined in this study. In other analyses, data from the 17 surveys were combined. These include the analyses of chronic disease and severe psychological distress. Data for the quit attempt rate and 3-month quit rate were also combined over the 17 years. This simplifies the tables, as preliminary analysis showed the data patterns for these measures were similar over the 17 years. For all analyses using combined data, 95% confidence intervals were computed. Statistical Analysis System (SAS) Version 9.4 was used for the analyses.[17]

## Results

**Fig 1:**
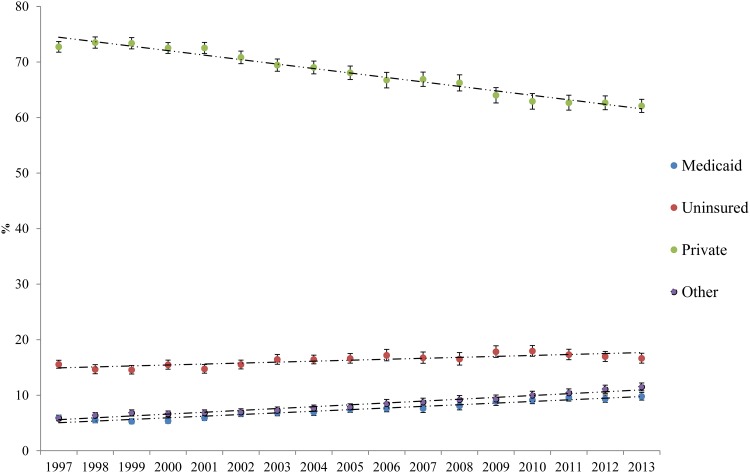
Proportion of US adults in each insurance category. Error bars represent the 99.7% confidence intervals, used to adjust for multiple comparisons (retaining an overall 95% family-wise error for the 17 surveys from 1997 to 2013).

Fig 1 shows the proportion of US adults in each insurance category by year. The proportion in Private Insurance declined over the study period, 1997–2013, while those in the other three insurance categories increased. The proportion in Medicaid nearly doubled from about 5% to about 10% of adults.

Table 1 shows the demographic breakdown of US adults by insurance category, averaged over the 17-year study period. It also presents breakdowns for the first and last years of the study to show changes over time. Corresponding to the large shifts in insurance coverage shown in Fig 1, there were large demographic shifts within groups. The proportion of female Medicaid recipients decreased from 70.7% in 1997 to 62.6% in 2013. The proportion with less than a high school education decreased from 48.7% to 34.5%. Proportions of non-Hispanic Whites and Blacks decreased while those of Hispanics and Others increased.

**Table 1:** Demographics of US adults by insurance coverage (average over 17 surveys and range from 1997–2013).

Characteristic	All				Medicaid				Uninsured				Private				Other			
	N	%	1997	2013	N	%	1997	2013	N	%	1997	2013	N	%	1997	2013	N	%	1997	2013
**Gender**																				
** Female**	288644	**51.9**	52.0	51.8	34440	**66.3**	70.7	62.7	44570	**46.3**	46.3	47.0	181274	**51.7**	51.5	51.5	28360	**52.3**	54.2	51.6
** Male**	225399	**48.1**	48.0	48.2	13635	**33.7**	29.3	37.3	43730	**53.7**	53.7	53.0	146016	**48.4**	48.5	48.6	22018	**47.7**	45.8	48.4
**Age**																				
** 18–34**	149075	**31.2**	33.0	30.2	16596	**40.0**	40.9	39.6	41107	**50.3**	54.4	46.1	87673	**28.6**	29.6	28.7	3699	**7.6**	11.0	7.5
** 35–49**	147587	**29.7**	31.8	25.6	12027	**25.2**	24.8	23.0	29566	**31.5**	31.0	30.4	101495	**32.1**	34.2	28.0	4499	**9.8**	11.5	7.9
** 50–64**	115652	**22.5**	18.7	25.9	9689	**18.5**	15.4	22.6	16736	**17.2**	14.0	22.5	80851	**24.6**	20.2	28.6	8376	**17.7**	16.3	18.8
** 65+**	101729	**16.6**	16.4	18.3	9763	**16.0**	18.8	14.9	891	**0.8**	0.7	1.1	57271	**14.6**	16.0	14.7	33804	**64.9**	61.2	65.9
**Education**																				
** <12**	96494	**16.4**	19.6	13.8	20588	**40.8**	48.7	34.5	28112	**29.8**	33.2	26.0	33670	**9.5**	13.0	6.5	14124	**26.1**	37.1	18.3
** = 12**	142577	**28.8**	30.3	26.0	14204	**32.0**	29.2	31.8	27298	**33.6**	32.7	32.4	86042	**27.1**	29.8	22.5	15033	**31.4**	30.2	31.3
** Some college**	146933	**29.4**	28.4	30.9	9902	**20.7**	17.6	25.5	22739	**26.5**	25.8	29.2	101476	**31.3**	30.3	32.5	12816	**26.2**	22.3	29.5
** BA +**	123709	**25.3**	21.8	29.3	2709	**6.2**	4.6	8.2	9086	**10.2**	8.3	12.4	104238	**31.9**	27.0	38.6	7676	**16.2**	10.5	20.9
**Race**																				
** NH-White**	322458	**70.8**	74.6	66.1	18295	**48.5**	49.6	45.8	36503	**50.8**	57.6	44.9	235409	**77.7**	80.5	73.9	32251	**72.2**	71.8	72.2
** NH-Black**	73530	**11.2**	11.0	11.5	13066	**23.3**	26.0	22.2	14169	**14.2**	13.6	14.8	37381	**8.9**	8.8	9.0	8914	**13.4**	14.9	11.6
** Hispanic**	88332	**12.3**	9.9	15.0	13551	**20.9**	18.3	21.9	32440	**28.7**	22.5	33.7	35772	**7.8**	6.6	9.9	6569	**9.4**	8.8	10.1
** Other**	29642	**5.7**	4.5	7.3	3162	**6.9**	6.1	10.1	5174	**6.4**	6.3	6.6	18668	**5.5**	4.1	7.3	2638	**5.0**	4.5	6.2

Note: NH stands for Non-Hispanic

The changing proportion of US adults covered by Medicaid, and the changing demographics of those covered, make it necessary to adjust the estimates of smoking prevalence when comparing across multiple years. For example, Hispanics tend to have a lower smoking prevalence. An increased proportion of Hispanics in Medicaid over time means the smoking prevalence for Medicaid will decrease even if no one actually quits smoking.

**Fig 2:**
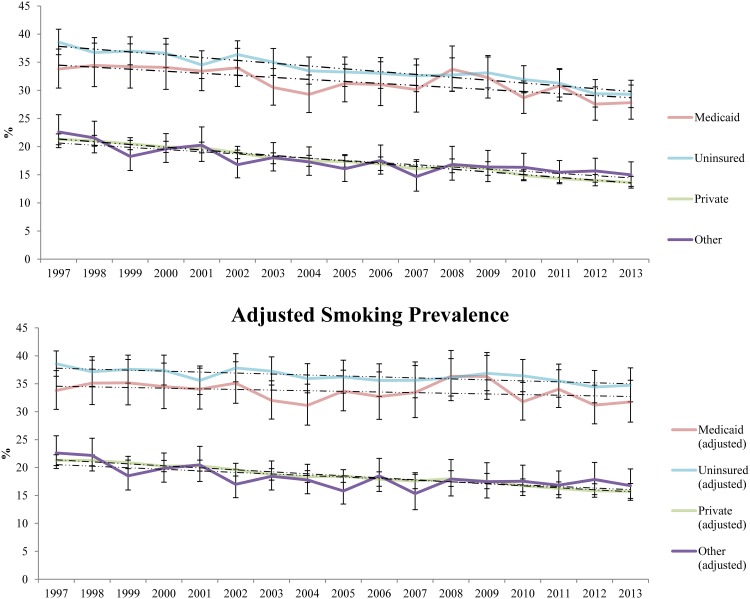
Smoking prevalence of US adults by insurance coverage. The top panel: Error bars represent the 99.7% confidence intervals, used to adjust for multiple comparisons (retaining an overall 95% family-wise error for the 17 surveys from 1997 to 2013). The bottom panel: Error bars represent the 99.7% confidence intervals, used to adjust for multiple comparisons (retaining an overall 95% family-wise error rate for the 17 surveys from 1997 to 2013). Data from the 1998 and the later surveys were also re-weighted to the demographic composition (i.e. gender, age, education, race/ethnicity) of the 1997 survey.

Fig 2 shows smoking prevalence rates by year from 1997 to 2013. The top panel presents crude rates based on the sampling weights of each NHIS survey year. The bottom panel presents adjusted estimates, standardized according to the demographics of 1997.

The top panel shows that from 1997 to 2013, there was an apparent decrease in the unadjusted smoking prevalence rates across all four insurance categories. Rates decreased from 33.8% to 27.8% in Medicaid, from 38.6% to 29.3% in the Uninsured, from 21.3% to 13.7% in Private Insurance, and from 22.6% to 15.0% in Other Coverage. A liberal test for trends found significant linear trends for all four groups (all *P*’s<0.001). A conservative test, in which 99.7% confidence intervals were used (as plotted in Fig 2), shows that all confidence intervals for Medicaid are overlapping, indicating that the decrease in that group is not significant, whereas each of the other three insurance categories has non-overlapping confidence intervals, indicating that decreases in those groups are significant.

The bottom panel shows adjusted smoking prevalence rates over the same 17-year period. As with the unadjusted rates, there was an apparent decrease in smoking prevalence in all four groups: from 33.8% to 31.8% in Medicaid, from 38.6% to 34.7% in the Uninsured, from 21.3% to 15.8% in Private Insurance, and from 22.6% to 16.8% in Other Coverage. However, even a liberal test for trends found that the change in Medicaid was not significant (*P* = 0.13). A conservative test, in which 99.7% confidence intervals were used (as plotted in Fig 2), shows large overlaps between the confidence intervals for smoking prevalence in the Medicaid population. In contrast, trends for the three other groups remained significant after adjustment.

The fact that smoking prevalence has declined in all insurance group except Medicaid, while the proportion of the population insured by Medicaid has increased, means that the proportion of US adult smokers in Medicaid has also increased over time. In fact, from 1997 to 2013, the proportion of US adult smokers in Medicaid more than doubled from 8.0% to 17.1%.

Table 2 shows quit ratios assessed during the last survey, in 2013. Quit ratio, the proportion of ever smokers who have quit at the time of the survey, generally increases with age, a trend seen in all four insurance categories. However, there are important intergroup differences. Those in Medicaid are less likely to have quit than those in Private Insurance, both overall and in every age bracket. Even in the oldest bracket, 65 and older, when most ever smokers would have quit smoking or deceased, there is still a significant difference between Medicaid and Private Insurance. Quit ratios for Medicaid are also lower than for Other Coverage, both overall and in half of the age brackets. In contrast, quit ratios for the Uninsured track those of Medicaid closely. Overall, 38.8% of ever smokers in Medicaid had quit smoking by 2013, compared to 32.0% for the Uninsured, 62.3% for Private Insurance, and 69.8% for Other Coverage (all *P’s*<0.01).

**Table 2:** Quit ratio by age and insurance coverage (2013).

Insurance	All		18–34		35–49		50–64		65+	
	N	%(95%CI)	N	%(95%CI)	N	%(95%CI)	N	%(95%CI)	N	%(95%CI)
Medicaid	1866	**38.8** (35.9–41.9)	461	**23.5** (19.1–28.7)	448	**26.6** (20.9–33.1)	597	**48.2** (42.7–53.6)	360	**71.7** (65.5–77.2)
Uninsured	2557	**32.0** (29.6–34.6)	924	**24.5** (21.1–28.3)	849	**32.5** (28.3–37.0)	760	**41.3** (37.2–45.6)	24	**66.7** (42.4–84.4)
Private	7146	**62.3** (60.7–63.9)	1401	**42.6** (39.2–46.0)	1689	**57.3** (54.1–60.4)	2294	**64.2** (61.7–66.6)	1762	**85.5** (83.2–87.6)
Other	2267	**69.8** (67.1–72.4)	97	**31.9** (20.8–45.6)	141	**52.3** (41.6–62.9)	488	**53.7** (48.0–59.4)	1541	**80.0** (77.4–82.4)
All	13836	**55.3** (54.0–56.5)	2883	**33.8** (31.8–35.9)	3127	**47.6** (45.2–50.0)	4139	**57.5** (55.6–59.5)	3687	**82.1** (80.4–83.7)

Note: Confidence intervals in the table are not adjusted for multiple comparisons

**Fig 3:**
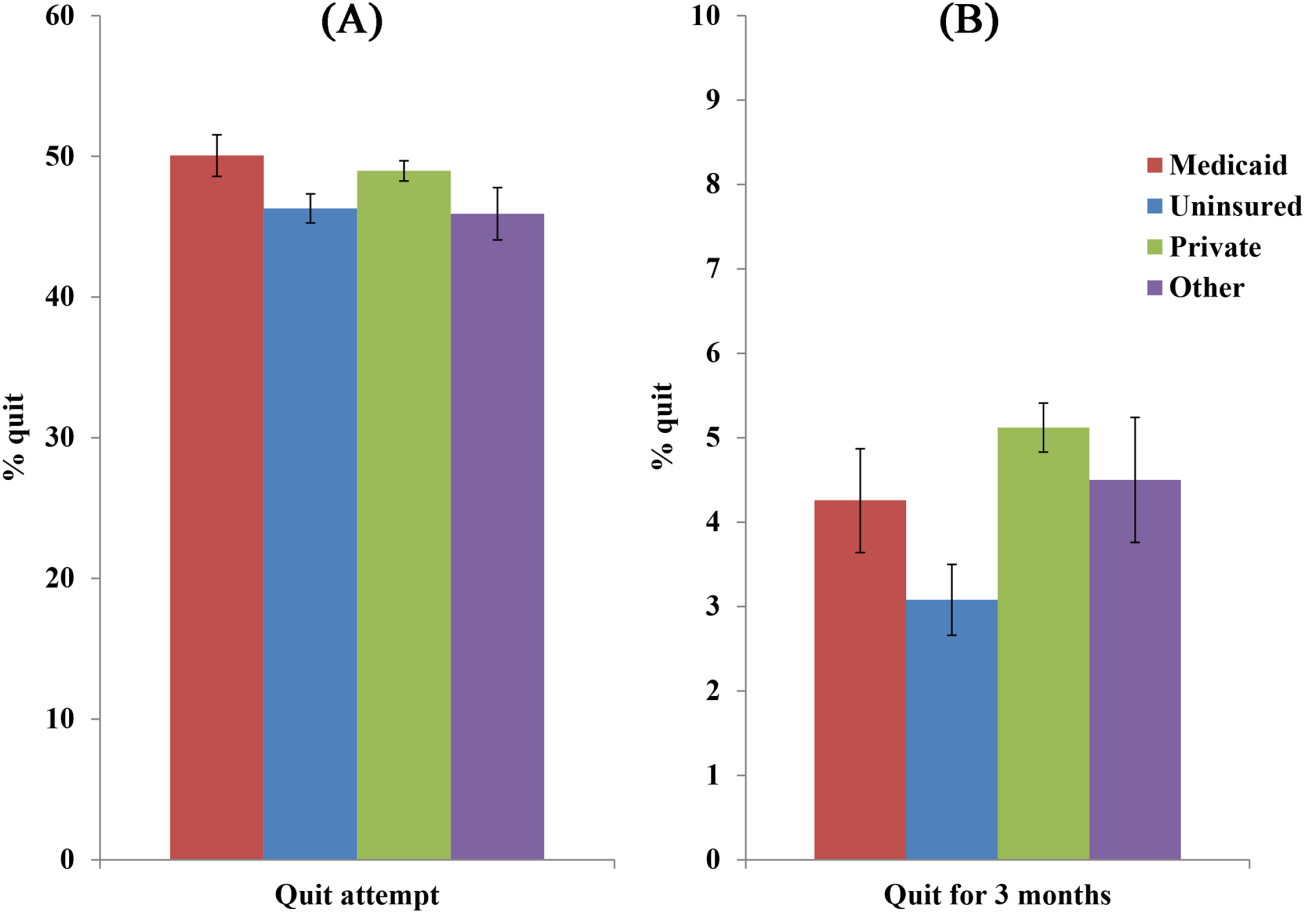
Quit attempt rate and 3-month quit rate by insurance coverage. (A) The average quit attempt rate among those currently smoking or smoking in the past year from 1997 to 2013. Error bars represent the 95% confidence intervals. The weights were also adjusted to the sum of the observed sample size of each survey. (B) The average annual quit rate among those currently smoking or smoking in the past year from 1997 to 2013. Error bars represent the 95% confidence intervals. The weights were also adjusted to the sum of the observed sample size of each survey.

Fig 3 shows annual rates of making a quit attempt, averaged over the 17 surveys, among those currently smoking or smoking within the past year, by insurance category. It indicates that those in Medicaid are as likely as those in Private Insurance to make a quit attempt (50.1% for Medicaid vs 49.0% for Private Insurance; *P* = 0.19), and more likely than the Uninsured and Other Coverage (46.3% and 45.9%, respectively; both *P*’s<0.001 for the comparisons to Medicaid).

Fig 3 also shows average annual rates of quitting for 3 months or more. It indicates that those in Medicaid are less likely than those in Private Insurance to have quit for at least 3 months (4.3% for Medicaid vs 5.1% for Private Insurance; *P* = 0.026), as likely as those in Other Coverage (4.5%; *P* = 0.62), and more likely than the Uninsured (3.1%; *P*<0.005).

Table 3 shows the average annual rates at which current smokers report ever having been diagnosed with hypertension, heart disease, stroke, emphysema, asthma, cancer, or diabetes, by insurance category. In general, those in Other Coverage—mostly Medicare seniors—have the highest rates. Among the other groups, the rates of all seven conditions are significantly higher in Medicaid than in either Private Insurance or the Uninsured. The combined rate of smokers reporting any of the seven conditions is 55.0% in Medicaid, compared to 37.3% in Private Insurance and 32.4% in the Uninsured (both *P*’s<0.01). For smokers with at least one condition, the mean number of conditions (not shown) is 1.8 for Medicaid, compared to 1.4 for Private Insurance and 1.4 for the Uninsured (both *P*’s<0.01).

**Table 3:** Chronic diseases conditions among current smokers (averaged over 17 surveys).

Condition	Medicaid	Uninsured	Private	Other
	N = 14,416	N = 27,998	N = 57,166	N = 8,731
	% (95% CI)	% (95% CI)	% (95% CI)	% (95% CI)
Hypertension	**31.5** (28.7–34.3)	**16.2** (14.3–18.1)	**20.8** (19.5–22.1)	**45.5** (42.0–49.0)
Heart disease	**17.3** (16.2–18.3)	**7.0** (6.3–7.7)	**8.9** (8.4–9.4)	**23.4** (22.1–24.7)
Stroke	**5.2** (4.8–5.5)	**1.2** (1.0–1.5)	**1.6** (1.4–1.8)	**8.1** (7.7–8.6)
Emphysema	**7.0** (6.6–7.5)	**1.6** (1.3–2.0)	**2.2** (2.0–2.4)	**10.0** (9.4–10.6)
Asthma	**22.1** (20.6–23.6)	**11.8** (10.6–12.9)	**10.1** (9.3–10.8)	**12.8** (10.9–14.7)
Cancer	**8.2** (7.2–9.2)	**3.6** (2.9–4.2)	**5.6** (5.1–6.0)	**13.5** (12.2–14.7)
Diabetes	**10.1** (8.7–11.5)	**3.5** (2.6–4.5)	**4.8** (4.1–5.5)	**12.8** (11.1–14.6)
Any disease	**55.0** (52.0–58.1)	**32.4** (30.3–34.5)	**37.3** (35.9–38.8)	**65.5** (61.6–69.5)
18–34	**37.2** (34.5–39.8)	**24.3** (22.6–25.9)	**23.6** (22.2–24.9)	**34.5** (28.3–40.7)
35–49	**56.4** (53.7–59.2)	**35.0** (33.2–36.8)	**32.4** (31.2–33.6)	**52.4** (47.5–57.4)
50–64	**79.5** (76.3–82.7)	**51.0** (48.5–53.5)	**53.1** (51.6–54.5)	**74.0** (70.5–77.5)
65+	**82.1** (77.6–86.7)	**47.8** (33.0–62.6)	**72.0** (69.5–74.5)	**73.2** (70.3–76.1)

Note: Confidence intervals in the table are not adjusted for multiple comparisons.

Table 3 also shows the rate of reporting any of the seven conditions by four age brackets, since disease prevalence is highly correlated with age. In each age bracket, smokers with Medicaid are significantly more likely than those in Private Insurance or the Uninsured to have one of the seven conditions.

Table 4 shows average annual rates of severe psychological distress among adult US smokers. At 16.2%, smokers with Medicaid have the highest rate of distress of all four insurance groups. They are more than twice as likely as the Uninsured, at 7.6%, and five times as likely as those in Private Insurance, at 3.2%, to report distress (both *P*’s<0.001). The rates are also broken down by gender because women in general report mental illness at higher rates than men.[18,19] In both women and men, smokers with Medicaid are significantly more likely to report psychological distress than their counterparts in all other insurance groups.

**Table 4:** Severe psychological distresses among current smokers (averaged over 17 surveys).

Gender	N	Medicaid % (95%CI)	N	Uninsured % (95%CI)	N	Private % (95%CI)	N	Other % (95%CI)
Women	9,550	**16.7** (16.0–17.4)	11,894	**10.4** (9.7–11.0)	28,221	**4.4** (4.0–4.8)	3,894	**10.1** (9.0–11.1)
Men	4,663	**15.1** (14.0–16.1)	15,823	**5.7** (5.1–6.2)	28,561	**2.1** (1.7–2.4)	4,731	**9.7** (8.7–10.7)
Mean	14,213	**16.2** (15.5–16.9)	27,717	**7.6** (7.1–8.0)	56,782	**3.2** (2.9–3.5)	8,625	**9.9** (9.1–10.8)

Note: Confidence intervals in the table are not adjusted for multiple comparisons.

## Discussion

### Medicaid tends to “collect” smokers

Previous research has found that the low-income population in the US has a much higher rate of smoking than that of the higher income population, and that it is declining more slowly.[1,3] The current study found a striking difference in the rates of decline in smoking prevalence between those with and without Medicaid in the 17 years prior to the expansion of Medicaid in 2014 under the Affordable Care Act (ACA). Since Medicaid is the primary means-tested form of insurance in the US, it is not surprising that smoking prevalence declines more slowly in Medicaid than in the non-Medicaid population. However, data from NHIS, the largest health survey in the US, indicate that the change in smoking prevalence for Medicaid is so slow as to be statistically negligible (Fig 2, second panel).

The reasons for this lack of change are not obvious, but certain explanations can be ruled out. First, there is no floor effect at work in these data. About 28% of the adult Medicaid population smoke, much higher than the rate of the overall adult population. In states where there are strong tobacco control programs, smoking prevalence for Medicaid recipients is significantly lower. For example, the 2014 smoking prevalence for Medicaid in California was 14.7%.[20] There is clearly ample room to decrease the smoking prevalence among Medicaid recipients nationally.

Second, it should be noted that NHIS is not a longitudinal survey, so there is no single cohort of Medicaid recipients who simply failed to quit smoking over the 17-year study period. Indeed, since the average recipient retains coverage for only about 3 years there was likely substantial turnover in the Medicaid group during the study period.[21] The relatively unchanging prevalence rates in Medicaid suggest that any smoker who quit must have been replaced by a “new” smoker joining Medicaid.

Taken together with the fact that smoking prevalence did drop significantly in all other insurance categories, this suggests that Medicaid tends to “collect” smokers over time. Smokers generally have to make repeated attempts to quit before succeeding for good. It appears that, during the time when smokers are receiving Medicaid, they are not succeeding in quitting. They may succeed in their next attempt, but by then they may have left Medicaid. In other words, there is connection between being covered by Medicaid and having difficulties in life, including difficulty in quitting smoking. In fact, with Medicaid enrollment rising, smokers have become increasingly concentrated in the Medicaid population. In 1997 they represented 8.0% of all adult smokers in the US, but by 2013—just before the ACA expansion of Medicaid—that figure had more than doubled to 17.1%. This upward trend will likely continue if Medicaid continues to expand.

### Smokers in medicaid try to quit but have less success

Regardless of how Medicaid became the primary carrier of a large number of smokers, one thing is clear from this study: those in Medicaid are far less likely than their counterparts in other insurance groups to have quit smoking (Table 2). Only 38.8% of ever smokers in Medicaid had quit smoking at the time of the 2013 survey, compared to 62.3% of the privately insured and 69.8% of those with other types of coverage. Only the uninsured have a similarly low quit ratio, 32.0%.

It should be noted that while young smokers were more likely to try to quit smoking than older smokers in any given year,[22] most smokers must make repeated quit attempts before they quit for good.[23] As a result, young smokers might have a lower quit ratio because they have had fewer opportunities to try to quit. Since Medicaid smokers tended to be younger than smokers covered by private insurance, Table 2 presents the data by age group. The results showed Medicaid smokers in each age group had lower quit ratios than those covered by private insurance plans.

Yet smokers in Medicaid are not less likely than others to try to quit in any given year. In fact, each year on average approximately half of Medicaid smokers, 50.1%, make a quit attempt. This rate is statistically equivalent to that of the privately insured (49.0%), and actually higher than the rates of the uninsured and those with other coverage (46.3% and 45.9%, respectively).

The problem, as may be inferred from the high quit attempt rate and low quit ratio, is the greater risk of relapse among Medicaid members. This is borne out by the fact that the average 3-month quit rate (a measure of annual cessation) is only 4.3% in Medicaid, compared to 5.1% among the privately insured.

That Medicaid smokers were no less likely to try to quit but were less likely to succeed in their attempt supports previous research from England on the association of social gradient and smoking cessation.[24] Smokers of lower income and social standing face more challenges in life than other smokers. As shown in Table 4, they tend to experience a greater level of psychological distress in daily life. As a result, their chance of success is further reduced in trying to quit, a task that is already very difficult for most.

On the other hand, the fact that Medicaid smokers were no less likely to try to quit than those covered by private insurance is an encouraging sign. Typically, lower socioeconomic status is associated with both a lower rate of attempt and a lower probability of success per quit attempt.[25] That Medicaid smokers are motivated to change their health behavior suggests that they may be responsive to interventions aiming to help them succeed in quitting.

### Smokers in medicaid face significant challenges

In addition to looking at differences in tobacco use and cessation among the four insurance groups, this study also examined the NHIS data for differences in physical and mental health. Smokers in Medicaid are far more likely than those with private insurance or the uninsured to suffer chronic disease. Each of seven common conditions—hypertension, heart disease, stroke, emphysema, asthma, cancer, and diabetes—is much more prevalent among Medicaid smokers than among smokers in the other two groups. Individuals with private insurance are likely to have higher socioeconomic status, which would predict a lower prevalence of chronic disease.[26] Similarly, the uninsured include many young and comparatively healthy individuals, which would explain why their health profile is more favorable than that of Medicaid beneficiaries. Moreover, the uninsured who develop significant health conditions (whether acute or chronic) may enroll in Medicaid.

With its high rates of smoking and chronic disease, Medicaid accounts for a large and growing share of the smokers with chronic disease. For example, in the last year of this study (2013), 21.5% of US adult smokers with chronic disease were insured through Medicaid, even though only 9.8% of the entire adult population was in the program.

The survey data also reveal important differences with respect to mental health. Nearly 1 in 6 Medicaid smokers, 16.2%, report experiencing severe psychological distress. Many more experience distress that is not severe but is still associated with elevated smoking and reduced cessation.[27] The rate of severe distress among smokers in Medicaid is several times higher than that of the privately insured (3.2%), and more than twice that of the uninsured (7.6%). Given that the K6 scale used in the survey to assess non-specific psychological distress is strongly associated with current mental health diagnoses,[15] these findings suggest that there is a large number of smokers in Medicaid, growing over time as a proportion of total smokers, who have mental health conditions. Such conditions make behavior change more difficult, and increase the need for active assistance to quit.[28,29]

## Conclusions

This study has some important limitations. Since NHIS is a periodic point in time survey with no carryover of survey respondents from year to year, and does not assess individual changes in insurance status, there is an unknown amount of mobility among the four insurance categories over time. Individuals may cycle in and out of Medicaid as their economic situation fluctuates, making it very difficult to relate population changes to changes on the individual level. Cross-sectional nature of the survey also makes it difficult to assess causality. Second, in order to have a string of surveys with comparable samples and methodology, we limited the study to survey years 1997, when the survey was revamped, through 2013, just before ACA expanded Medicaid. Future research is needed to examine whether and how the trends in tobacco cessation by insurance status changed following this expansion. Finally, the study only examined cigarette smoking. In the interest of space, the use of other tobacco products was not included in the analysis.

Notwithstanding these limitations, this study draws attention to some critical facts: that while progress has been made reducing overall smoking prevalence in the US, the Medicaid population still smokes at a high and essentially unchanging rate; that while smokers in Medicaid are as likely as others to try to quit, they have less success doing so; that they suffer much more chronic disease (due in part, no doubt, to their greater tobacco use); and that they are far more likely to experience severe psychological distress than their counterparts in other groups. This study also shows that the proportion of the nation’s smokers with comorbid conditions (whether physical or mental) who are insured by Medicaid is rapidly increasing. Medicaid is not just collecting smokers, but is collecting those smokers most likely to need help to quit.

These considerations strongly indicate that Medicaid programs should make tobacco control a top priority.[5] Even before the ACA expansion, Medicaid programs were where large and growing numbers of the country’s most vulnerable smokers were concentrated. The expansion presents an even greater opportunity to improve public health by using Medicaid programs to implement effective and comprehensive smoking cessation interventions.[30] An earlier example of Massachusetts presents a hopeful example of how changes in cessation policy may have an impact on smoking prevalence.[31,32] Future research should examine if implementation of Medicaid cessation policies related to ACA expansion is having a similar effect.

To maximize impact, a campaign to reduce tobacco use in Medicaid should be targeted, accounting for changes in the Medicaid smoker profile. It should be comprehensive, taking full advantage of all evidence-based strategies in the areas of policy, communications, and treatment. Moreover, it should be sustained over the long term, since most smokers must make multiple quit attempts before they finally quit for good. An ongoing effort would also account for the movement of individuals in and out of Medicaid, thereby reaching many more smokers than are covered by Medicaid in any one year. Over time, such an effort has the potential to greatly reduce the personal and financial costs of tobacco use in the US.[5]

## Supporting information

S1 File STROBE statement. (DOCX)
