# Supplementary material for: Smoking prevalence in Medicaid has been declining at a negligible rate
Source: PLoS One. 2017 May 25;12(5):e0178279. doi: 10.1371/journal.pone.0178279 (PMC5479677; doi:10.1371/journal.pone.0178279)
Supplement: S1 File — (DOCX) [file pone.0178279.s001.docx]

STROBE Statement—checklist of items that should be included in reports of observational studies

|  | Item No. | Recommendation | Page  No. | Relevant text from manuscript |
| --- | --- | --- | --- | --- |
| **Title and abstract** | 1 | (*a*) Indicate the study’s design with a commonly used term in the title or the abstract | 2 | Abstract |
|  |  | (*b*) Provide in the abstract an informative and balanced summary of what was done and what was found | 2 | Abstract |
| Introduction | | | |  |
| Background/rationale | 2 | Explain the scientific background and rationale for the investigation being reported | 3 | Introduction |
| Objectives | 3 | State specific objectives, including any prespecified hypotheses | 4-5 | The present study aims to assess progress made toward reducing the rate of smoking by Medicaid recipients prior to expansion. In order to accomplish this, the study examines National Health Interview Survey (NHIS) data collected from 1997, when the survey was redesigned, to 2013, just before the expansion. The NHIS is the most comprehensive source of information about the health of the US population, and because it assesses health insurance coverage as well as smoking behavior it is ideal for the purpose of analyzing patterns of tobacco use by insurance status.[13] This study compares the smoking behavior of those with and without Medicaid coverage. It also compares them with respect to the prevalence of common chronic diseases that may be caused or aggravated by smoking. To explore further the factors that may contribute to increased difficulty with quitting, the study also examines differences in rates of psychological distress. By understanding the patterns of tobacco use and cessation in the Medicaid population, stakeholders will be better equipped to help Medicaid members quit. |
| Methods | | | |  |
| Study design | 4 | Present key elements of study design early in the paper | 4-5 | The present study aims to assess progress made toward reducing the rate of smoking by Medicaid recipients prior to expansion. In order to accomplish this, the study examines National Health Interview Survey (NHIS) data collected from 1997, when the survey was redesigned, to 2013, just before the expansion. The NHIS is the most comprehensive source of information about the health of the US population, and because it assesses health insurance coverage as well as smoking behavior it is ideal for the purpose of analyzing patterns of tobacco use by insurance status.[13] This study compares the smoking behavior of those with and without Medicaid coverage. It also compares them with respect to the prevalence of common chronic diseases that may be caused or aggravated by smoking. To explore further the factors that may contribute to increased difficulty with quitting, the study also examines differences in rates of psychological distress. By understanding the patterns of tobacco use and cessation in the Medicaid population, stakeholders will be better equipped to help Medicaid members quit. |
| Setting | 5 | Describe the setting, locations, and relevant dates, including periods of recruitment, exposure, follow-up, and data collection | 5 | NHIS is a continuously administered in-person household interview survey conducted by the CDC’s National Center for Health Statistics.[13] NHIS data were selected starting in 1997, when the survey underwent a major redesign. 2013 was chosen as the final study year because in 2014 the Affordable Care Act (ACA) began sharply expanding Medicaid. The combined sample size of the 17 annual surveys used in the study is 514,043. Survey methods can be found at http://www.cdc.gov/nchs/nhis/methods.htm. |
| Participants | 6 | (*a*) *Cohort study*—Give the eligibility criteria, and the sources and methods of selection of participants. Describe methods of follow-up  *Case-control study*—Give the eligibility criteria, and the sources and methods of case ascertainment and control selection. Give the rationale for the choice of cases and controls  *Cross-sectional study*—Give the eligibility criteria, and the sources and methods of selection of participants | 5 | NHIS is a continuously administered in-person household interview survey conducted by the CDC’s National Center for Health Statistics.[13] NHIS data were selected starting in 1997, when the survey underwent a major redesign. 2013 was chosen as the final study year because in 2014 the Affordable Care Act (ACA) began sharply expanding Medicaid. The combined sample size of the 17 annual surveys used in the study is 514,043. Survey methods can be found at http://www.cdc.gov/nchs/nhis/methods.htm. |
|  |  | (*b*) *Cohort study*—For matched studies, give matching criteria and number of exposed and unexposed  *Case-control study*—For matched studies, give matching criteria and the number of controls per case | NA |  |
| Variables | 7 | Clearly define all outcomes, exposures, predictors, potential confounders, and effect modifiers. Give diagnostic criteria, if applicable | 5-7 | **Measures of Smoking and Quitting**  Ever smokers are those who have smoked 100 or more cigarettes in their lifetimes. Current smokers are those ever smokers who smoked cigarettes every day or some days at the time of survey. Smoking prevalence is defined as the percentage of adults who are current smokers at the time of the interview. The quit ratio is the percentage of ever smokers who report at the time of the survey that they no longer smoke.  The quit attempt rate is the percentage of smokers who made a quit attempt in the previous 12 months. A quit attempt is an intentional cessation of tobacco use for at least 24 hours. The 3-month quit rate is the percentage of smokers who tried to quit smoking in the last 12 months and succeeded in quitting for at least 3 months at the time of survey.  **Measure of Insurance Status**  Health insurance status in the NHIS data is coded in four categories: Medicaid, Uninsured, Private Insurance, and Other Coverage. Medicaid includes those who do not have private insurance but who do have Medicaid or another state-sponsored health plan. Uninsured includes those who have no health insurance, who only have the Indian Health Service, or who only have a plan that pays for a single type of service such as dental care. Private Insurance includes those with a comprehensive insurance plan provided by an employer, purchased directly, or obtained through local or community programs. Other Coverage includes those with Medicare, a military health plan such as TRICARE, VA, or CHAMPVA, or government-provided coverage other than Medicaid.[14]  **Measures of Physical and Psychological Health**  Chronic diseases studied include hypertension, heart disease, stroke, emphysema, asthma, cancer and diabetes. Subjects are considered to have chronic disease if they report that a health professional ever told them they have one of the above conditions. Heart disease is a composite of four conditions assessed individually in the survey: coronary heart disease, angina pectoris, heart attack, and other heart condition or disease.  Subjects are considered to be experiencing severe psychological distress if they score 13 or higher on an NHIS question that is based on the Kessler Psychological Distress Scale or K6.[15] Subjects are asked, “During the past 30 days, about how often did you feel (1) nervous, (2) hopeless, (3) restless or fidgety, (4) so sad or depressed that nothing could cheer you up, (5) that everything was an effort, [or] (6) worthless?” Responses are coded as 4=all of the time, 3=most of the time, 2=some of the time, 1=a little of the time, or 0=none of the time. |
| Data sources/ measurement | 8* | For each variable of interest, give sources of data and details of methods of assessment (measurement). Describe comparability of assessment methods if there is more than one group | 5-7 | **Measures of Smoking and Quitting**  Ever smokers are those who have smoked 100 or more cigarettes in their lifetimes. Current smokers are those ever smokers who smoked cigarettes every day or some days at the time of survey. Smoking prevalence is defined as the percentage of adults who are current smokers at the time of the interview. The quit ratio is the percentage of ever smokers who report at the time of the survey that they no longer smoke.  The quit attempt rate is the percentage of smokers who made a quit attempt in the previous 12 months. A quit attempt is an intentional cessation of tobacco use for at least 24 hours. The 3-month quit rate is the percentage of smokers who tried to quit smoking in the last 12 months and succeeded in quitting for at least 3 months at the time of survey.  **Measure of Insurance Status**  Health insurance status in the NHIS data is coded in four categories: Medicaid, Uninsured, Private Insurance, and Other Coverage. Medicaid includes those who do not have private insurance but who do have Medicaid or another state-sponsored health plan. Uninsured includes those who have no health insurance, who only have the Indian Health Service, or who only have a plan that pays for a single type of service such as dental care. Private Insurance includes those with a comprehensive insurance plan provided by an employer, purchased directly, or obtained through local or community programs. Other Coverage includes those with Medicare, a military health plan such as TRICARE, VA, or CHAMPVA, or government-provided coverage other than Medicaid.[14]  **Measures of Physical and Psychological Health**  Chronic diseases studied include hypertension, heart disease, stroke, emphysema, asthma, cancer and diabetes. Subjects are considered to have chronic disease if they report that a health professional ever told them they have one of the above conditions. Heart disease is a composite of four conditions assessed individually in the survey: coronary heart disease, angina pectoris, heart attack, and other heart condition or disease.  Subjects are considered to be experiencing severe psychological distress if they score 13 or higher on an NHIS question that is based on the Kessler Psychological Distress Scale or K6.[15] Subjects are asked, “During the past 30 days, about how often did you feel (1) nervous, (2) hopeless, (3) restless or fidgety, (4) so sad or depressed that nothing could cheer you up, (5) that everything was an effort, [or] (6) worthless?” Responses are coded as 4=all of the time, 3=most of the time, 2=some of the time, 1=a little of the time, or 0=none of the time. |
| Bias | 9 | Describe any efforts to address potential sources of bias | N/A |  |
| Study size | 10 | Explain how the study size was arrived at | 5 | NHIS is a continuously administered in-person household interview survey conducted by the CDC’s National Center for Health Statistics.[13] NHIS data were selected starting in 1997, when the survey underwent a major redesign. 2013 was chosen as the final study year because in 2014 the Affordable Care Act (ACA) began sharply expanding Medicaid. The combined sample size of the 17 annual surveys used in the study is 514,043. Survey methods can be found at http://www.cdc.gov/nchs/nhis/methods.htm. |

Continued on next page

| Quantitative variables | 11 | Explain how quantitative variables were handled in the analyses. If applicable, describe which groupings were chosen and why | 5-7 | Health insurance status in the NHIS data is coded in four categories: Medicaid, Uninsured, Private Insurance, and Other Coverage. Medicaid includes those who do not have private insurance but who do have Medicaid or another state-sponsored health plan. Uninsured includes those who have no health insurance, who only have the Indian Health Service, or who only have a plan that pays for a single type of service such as dental care. Private Insurance includes those with a comprehensive insurance plan provided by an employer, purchased directly, or obtained through local or community programs. Other Coverage includes those with Medicare, a military health plan such as TRICARE, VA, or CHAMPVA, or government-provided coverage other than Medicaid.[14]  Chronic diseases studied include hypertension, heart disease, stroke, emphysema, asthma, cancer and diabetes. Subjects are considered to have chronic disease if they report that a health professional ever told them they have one of the above conditions. Heart disease is a composite of four conditions assessed individually in the survey: coronary heart disease, angina pectoris, heart attack, and other heart condition or disease.  Subjects are considered to be experiencing severe psychological distress if they score 13 or higher on an NHIS question that is based on the Kessler Psychological Distress Scale or K6.[15] Subjects are asked, “During the past 30 days, about how often did you feel (1) nervous, (2) hopeless, (3) restless or fidgety, (4) so sad or depressed that nothing could cheer you up, (5) that everything was an effort, [or] (6) worthless?” Responses are coded as 4=all of the time, 3=most of the time, 2=some of the time, 1=a little of the time, or 0=none of the time. |
| --- | --- | --- | --- | --- |
| Statistical methods | 12 | (*a*) Describe all statistical methods, including those used to control for confounding | 7-8 | For each individual survey year, all analyses were weighted to adjust for the unequal probability of selection in sampling. For this purpose, the weights provided by NHIS data set for each survey year were used.[16] When comparing the prevalence rate over time, we standardized the data from the 1998 and later surveys to the demographic composition of the 1997 survey. This was to ensure that apparent changes in population smoking behavior were not due simply to demographic changes over time. When the data from 1997 to 2013 were combined in the same analysis (e.g., Figure 3), the weights were adjusted to the sum of the observed sample size of each survey.  When testing for a trend of changing smoking prevalence over time, we used two methods: a liberal test in which a simple linear trend test was performed without considering increased type I error due to comparisons over multiple years, and a conservative test in which 99.7% confidence intervals, instead of 95% confidence intervals, were computed for each survey. The stricter confidence intervals were used to adjust for multiple comparisons, so as to retain an overall 95% family-wise error rate for the 17 surveys.  The quit ratio was analyzed for the last year of the survey examined in this study. In other analyses, data from the 17 surveys were combined. These include the analyses of chronic disease and severe psychological distress. Data for the quit attempt rate and 3-month quit rate were also combined over the 17 years. This simplifies the tables, as preliminary analysis showed the data patterns for these measures were similar over the 17 years. For all analyses using combined data, 95% confidence intervals were computed. Statistical Analysis System (SAS) Version 9.4 was used for the analyses.[17] |
|  |  | (*b*) Describe any methods used to examine subgroups and interactions | N/A |  |
|  |  | (*c*) Explain how missing data were addressed | N/A |  |
|  |  | (*d*) *Cohort study*—If applicable, explain how loss to follow-up was addressed  *Case-control study*—If applicable, explain how matching of cases and controls was addressed  *Cross-sectional study*—If applicable, describe analytical methods taking account of sampling strategy | 5 | NHIS is a continuously administered in-person household interview survey conducted by the CDC’s National Center for Health Statistics.[13] NHIS data were selected starting in 1997, when the survey underwent a major redesign. 2013 was chosen as the final study year because in 2014 the Affordable Care Act (ACA) began sharply expanding Medicaid. The combined sample size of the 17 annual surveys used in the study is 514,043. Survey methods can be found at http://www.cdc.gov/nchs/nhis/methods.htm. |
|  |  | (*e*) Describe any sensitivity analyses | N/A |  |
| Results | | | | |
| Participants | 13* | (a) Report numbers of individuals at each stage of study—eg numbers potentially eligible, examined for eligibility, confirmed eligible, included in the study, completing follow-up, and analysed | 5 | NHIS is a continuously administered in-person household interview survey conducted by the CDC’s National Center for Health Statistics.[13] NHIS data were selected starting in 1997, when the survey underwent a major redesign. 2013 was chosen as the final study year because in 2014 the Affordable Care Act (ACA) began sharply expanding Medicaid. The combined sample size of the 17 annual surveys used in the study is 514,043. Survey methods can be found at http://www.cdc.gov/nchs/nhis/methods.htm. |
|  |  | (b) Give reasons for non-participation at each stage | N/A |  |
|  |  | (c) Consider use of a flow diagram | N/A |  |
| Descriptive data | 14* | (a) Give characteristics of study participants (eg demographic, clinical, social) and information on exposures and potential confounders | 10 | Table 1 |
|  |  | (b) Indicate number of participants with missing data for each variable of interest | 10,13,17,18 | Tables 1-4 |
|  |  | (c) *Cohort study*—Summarise follow-up time (eg, average and total amount) | N/A |  |
| Outcome data | 15* | *Cohort study*—Report numbers of outcome events or summary measures over time | N/A |  |
|  |  | *Case-control study—*Report numbers in each exposure category, or summary measures of exposure | N/A |  |
|  |  | *Cross-sectional study—*Report numbers of outcome events or summary measures | 10,13,17,18 | Tables 1-4 |
| Main results | 16 | (*a*) Give unadjusted estimates and, if applicable, confounder-adjusted estimates and their precision (eg, 95% confidence interval). Make clear which confounders were adjusted for and why they were included | 10,13,17,18 | Tables 1-4, Figures 1-3 |
|  |  | (*b*) Report category boundaries when continuous variables were categorized | 5-7 | Health insurance status in the NHIS data is coded in four categories: Medicaid, Uninsured, Private Insurance, and Other Coverage. Medicaid includes those who do not have private insurance but who do have Medicaid or another state-sponsored health plan. Uninsured includes those who have no health insurance, who only have the Indian Health Service, or who only have a plan that pays for a single type of service such as dental care. Private Insurance includes those with a comprehensive insurance plan provided by an employer, purchased directly, or obtained through local or community programs. Other Coverage includes those with Medicare, a military health plan such as TRICARE, VA, or CHAMPVA, or government-provided coverage other than Medicaid.[14]  Chronic diseases studied include hypertension, heart disease, stroke, emphysema, asthma, cancer and diabetes. Subjects are considered to have chronic disease if they report that a health professional ever told them they have one of the above conditions. Heart disease is a composite of four conditions assessed individually in the survey: coronary heart disease, angina pectoris, heart attack, and other heart condition or disease.  Subjects are considered to be experiencing severe psychological distress if they score 13 or higher on an NHIS question that is based on the Kessler Psychological Distress Scale or K6.[15] Subjects are asked, “During the past 30 days, about how often did you feel (1) nervous, (2) hopeless, (3) restless or fidgety, (4) so sad or depressed that nothing could cheer you up, (5) that everything was an effort, [or] (6) worthless?” Responses are coded as 4=all of the time, 3=most of the time, 2=some of the time, 1=a little of the time, or 0=none of the time. |
|  |  | (*c*) If relevant, consider translating estimates of relative risk into absolute risk for a meaningful time period | N/A |  |

Continued on next page

| Other analyses | 17 | Report other analyses done—eg analyses of subgroups and interactions, and sensitivity analyses | N/A |  |
| --- | --- | --- | --- | --- |
| Discussion | | | | |
| Key results | 18 | Summarise key results with reference to study objectives | 19-25 |  |
| Limitations | 19 | Discuss limitations of the study, taking into account sources of potential bias or imprecision. Discuss both direction and magnitude of any potential bias | 23-24 |  |
| Interpretation | 20 | Give a cautious overall interpretation of results considering objectives, limitations, multiplicity of analyses, results from similar studies, and other relevant evidence | 19-25 |  |
| Generalisability | 21 | Discuss the generalisability (external validity) of the study results | 23-25 |  |
| Other information | |  | | |
| Funding | 22 | Give the source of funding and the role of the funders for the present study and, if applicable, for the original study on which the present article is based | Metadata | Journal requested that funding not be mentioned in the manuscript. |

*Give information separately for cases and controls in case-control studies and, if applicable, for exposed and unexposed groups in cohort and cross-sectional studies.

**Note:** An Explanation and Elaboration article discusses each checklist item and gives methodological background and published examples of transparent reporting. The STROBE checklist is best used in conjunction with this article (freely available on the Web sites of PLoS Medicine at http://www.plosmedicine.org/, Annals of Internal Medicine at http://www.annals.org/, and Epidemiology at http://www.epidem.com/). Information on the STROBE Initiative is available at www.strobe-statement.org.
